# Hospital and Institutional News

**Published:** 1913-06-07

**Authors:** 


### HOSPITAL AND INSTITUTIONAL NEWS.

### THE BIRTHDAY HONOURS.

Several decorations of interest to the institutional world occur in the list of Birthday Honours issued on Tuesday last. A baronetcy has been conferred on Mr. Arbuthnot Lane, F.R.C.S., the well-known Guy's surgeon, who has been attending the Duchess of Connaught. Among the new knights are Mr. Thomas Evans Flitcroft, L.R.C.P., President of the Lancashire and Cheshire Branch of the British Medical Association and chairman of the statutory medical committee under the Insurance Act; Mr. Andrew John Horne, F.R.C.P., ex-President of the Royal College of Physicians, Ireland; Mr. Herbert Smalley, M.D., Medical Inspector of Local Prisons and Superintending Officer of Convict Prisons since 1897; and Mr. Robert Henry Woods, F.R.C.S., President of the Irish Medical Association, and ex-President of the Royal College of Surgeons, Ireland. A knighthood has also been conferred on Major Edward Scott Worthington, M.V.O., R.A.M.C., medical officer to the Duke of Connaught since 1911. Major Worthington has been in constant attendance on the Duchess during her illness. New Companions of the Bath include Deputy Surgeon-General William Maxwell Craig, M.B., and Surgeon-General Louis Edward Anderson, Deputy Director of Medical Services, Ireland. Turning now to the Indian Honours List, a Knight Commandership of the Bath is conferred on Surgeon-General Henry Hamilton, C.B., M.D., Indian Medical Service, retired; while the C.S.I. is given to an institutional manager, Lieutenant-Colonel Gerald Godfray Giffard, M.R.C.P., M.R.C.S., Indian Medical Service, Superintendent Government Maternity Hospital, Madras. The honour of Knight Bachelor has been conferred on Mr. Alexander MacCormick, M.D., of Sydney, New South Wales. The "Kaisar-i-Hind" medal for Public Service in India has been awarded to Lieutenant Colonel Patrick Balfour Haig, M.B., Indian Medical Service Agency Surgeon, Bhopal, Central India, and to the Rev. John Buchanan, B.A., M.D., Missionary, Amkiet, Central India.

### INTERESTING PRESENTATION AT ISLEWORTH INFIRMARY.

Dr. E. E. Norton, who has just retired from the post of medical superintendent at the Brentford Workhouse Infirmary, Workhouse and Schools, was a few days ago the recipient of valuable presents from past and present members of the staff. Under the chairmanship of Dr. W. H. Williams (chairman of the infirmary committee) a large and representative gathering assembled at Isleworth, and Miss Youlden, the matron, presented the retiring superintendent with a gold hunter watch and a Sheraton silver cabinet, accompanied by an illuminated address, the latter designed by a grateful patient at the infirmary. Dr. Norton, who has always been popular with the staff and patients, has been appointed one of the five tuberculosis medical officers for the county of Middlesex. Since his appointment at Isleworth ten years ago the work has been more than doubled, and the chairman of the infirmary committee was pleased to testify that "Dr. Norton had not only superintended the institution with conspicuous ability, but to the entire satisfaction of the Guardians." Dr. Norton's new appointment was announced in our columns almost simultaneously with the publication of his important series of articles on "Milk and Milk Contracts." These point to the interest which he has long felt in the new work now undertaken by him.

### THE COMMAND OF TERRITORIAL MEDICAL UNITS.

Hitherto the various field ambulances, mounted brigade ambulances, and general hospitals of the Territorial Force have been commanded by lieutenant-colonels selected for their appointments from the majors of the Force. The impression is now very generally held that the War Office intend for the future to appoint to those commands only ex-Regular R.A.M.C. officers; and that promotion to lieutenant-colonel will be, in consequence, almost abolished for Territorial medical officers pure and simple. If this resolution has been formed, and is to be acted upon, we feel bound to admit that it will probably improve the efficiency of many of the Territorial medical units. Now and then a practising doctor can be found who has sufficient leisure, energy, keenness, and military instincts to bring a medical unit to a really high level; but such men are rare, and there are not enough of them to go round. At the same time it is rather hard to deprive good keen Territorial officers of the chance of promotion to lieutenant-colonel, especially if the consultants on the *à la suite* staff of the general hospitals will still be able to reach that rank without doing anything whatever to deserve it. We note that Dr. Bruce Porter, one of the ablest practitioners in the West of London, has just been appointed to command a general hospital; as he was formerly an officer of the Regular Royal Army Medical Corps, the selection seems admirable.

### ARMY RECRUITS AND SWEDISH DRILL.

Now that orthopædics are beginning to be recognised in this country as deserving of careful study and a special department, to say nothing of the need of acquainting English medical men with Continental methods, tributes to the value of Swedish exercises and Swedish drill are increasingly frequent. Last Saturday Surgeon-General Sir Launcelot Gubbins, who presented the prizes at the gymnastic display of the Officers' Training Corps contingent at King's College School, remarked that in his individual experience, which extended to forty years, with the Army all over the world, he could say that since the introduction of Swedish drill the physique of recruits had increased 50 per cent. That is a far-reaching statement, and an unqualified one, which may serve to comfort us on the next occasion on which severe criticism of the physique of our classes from which recruits are drawn is made. The value of the exercises themselves can have no better acknowledgment.

### MR. DAWES' CHARGE OF SNOBBERY.

One of the considerations which has influenced and does still influence, medical men in regard to joining an Insurance panel is the possible effect upon their private practice. Many of them felt apprehensions on this point last winter, and many also have found such fears justified by the events of the last five months. When Mr. Dawes, M.P., was invited to take into consideration the fact that those who join the panel stand to lose some of their private patients all he could say was that the latter are snobs, and that if a panel doctor is good enough for him others ought to think the same. This childish reply is cold comfort to panel doctors who are losing their private patients, and in any case the charge of snobbery in this connection seems to us quite unjust and insupportable. A member of the public who pays a fee to a medical practitioner may legitimately take into account the sort of service he gets in return; and if he knows that the doctor is under contract to attend some thousands of insured persons, he may quite properly make certain deductions and act upon them by declining to consult that particular doctor. The deductions in question might be right or not, but there is nothing in the least snobbish about them. One of them might be that a doctor with a large amount of daily work amongst poor people runs a greater risk of conveying contagious disease from patient to patient than one whose practice is confined to paying patients. Another such deduction might be that a man who has to see fifty, sixty, or a hundred panel patients a day is likely to get into slovenly habits of diagnosis and treatment, and, further, is likely to have lost his freshness of mind and energy by the time he sees his other patients. That might be meticulous, but it is certainly not snobbish.

### THE PERMANENT ENDOWMENT OF A HOSPITAL.

The late Miss Dow, of Montreal, has left £20,000 for the permanent endowment of the hospital at Gujarat in India. The announcement of this bequest was made at the General Assembly of the Church of Scotland in Edinburgh last week.

### ST. BARTHOLOMEW'S HOSPITAL FINANCES.

The annual report of Mr. G. Acton-Davies, acting treasurer of St. Bartholomew's Hospital, contains a number of interesting items and statistics. The income of the charity last year was £83,951, as against £77,556 in 1911. This increase is, of course, gratifying; but it must be remarked that all but £600 of it is due to the special appeal, which produced £5,834. The expenditure was less than that of 1911 by £6,400; but even then was £2,450 greater than the income. This, however, is a great improvement on the previous year, when the hospital was £15,330 to the bad on the year's working. Nine-tenths of the huge cost of the out-patients' block has been defrayed. The finances of the new pathological block are not quite satisfactory, as only about half the cost has been raised so far. The average number of beds occupied daily was 566, a figure smaller than it would otherwise have been owing to the closing of one-third of the hospital for seven weeks for cleaning purposes. The City motor ambulances conveyed to the hospital 1,704 cases of illness or accident during the year.

### THE GROWTH OF CREMATION.

The popularity of cremation may be growing slowly, but it is growing steadily, to judge by the "Transactions" just issued by the Cremation Society of England, which state that 1,134 cremations took place in 1912, or 111 more than in the previous year. It should be remembered that there are thirteen crematoria in the country, five of which are municipal, and, as we have previously shown, the fees can be as low as £2 12s. 6d., which, quoting from memory, represents the cheapest cremation to be had—namely, at Ilford. By the way, the *Times*, with traditional lack of humour, stigmatised as singular the following plea of the Cremation Society for this mode of disposing of the body: it "removes the possibility of being buried alive." It does. That the *Times* does not see this is because it shares the popular delusion that the fear of being buried alive is the same thing as the fear of death or of dying. It is neither. Men fear to be buried alive because no test of death, except the signs of putrefaction, has been discovered of such accuracy as to prevent the possibility of coma being mistaken for death; and the idea of suffocation in a coffin is revolting to healthy-minded men and women. Cremation makes such a possibility impossible. Though it cannot prevent coma from being mistaken for death, it first doubles the precautions taken by necessitating two certificates of death, and then provides—should there have been an error, which is most unlikely—a death so speedy as hardly to excite fear. The fact that such a quick death is a possibility deceives thoughtless persons into imagining that cremation would only confirm a tragic error—but when errors are really tragic it is best for the subject of them to have them confirmed as swiftly and surely as possible. This, like the words life and death themselves, involves a contradiction in terms, and consequently excites a smile among the extremely academic who fancy we can escape such contradictions.

### COURTSHIPS AND VISITORS' ROOMS IN MENTAL HOSPITALS.

It has often been remarked how frequently the mentally afflicted intermarry, as if a family taint of this kind were a positive attraction to another labouring under the same stigma. One of the causes of this undoubtedly appears to be the occasion for courtships which the visiting rooms of our mental hospitals afford. In these rooms meet the relatives of patients in the institutions, and such meetings are often the occasion of an intimacy which ends in matrimony. Our object in recording a simple observation of this kind is not hastily to suggest the imposition of further restrictions upon visitors to mental hospitals nor to insist on the segregation of the sexes among them. The question of restricting the propagation of mentally afflicted persons is one too large to be treated summarily in this fashion. But it is curious and regrettable to note that the visitors' rooms in our mental hospitals indirectly help to recruit the number of the patients likely to be sent to the institutions.

### THE LAST OF THE RED CRESCENT UNITS.

The relief work of the British Red Crescent Society in Asia Minor is now summarised by the Secretary in a letter to the press, which states that the Society's hospital at San Stefano has now been closed, and that the hospital unit at Adrianople is the only one left at the seat of war for relief of the Turkish sick. Widows, orphans, and refugees, where necessary, are still benefiting through the Anglo-Ottoman Relief Committee, which is carrying on its work as far as funds permit. "The medical officers of the British Red Crescent Mission in Adrianople, Dr. W. E. Haigh, Dr. Calthrop, Dr. Bayliss, and Dr. Speller, with one orderly are," reports the Secretary, "looking after the Turkish sick and wounded, numbering several thousands, in conjunction with the Bulgarian and Turkish doctors, who, however, are expected to leave shortly, on the conclusion of peace." The Society has already distributed £24,000 through its various agents, and not its least important work has been that concerned with assisting the dependants of the soldiers.

### THE WATER BOARD AND HOSPITAL EXTRAVAGANCE.

Attention to the loss caused by the increasing consumption of water at voluntary hospitals was called by the appeal and assessment committee at a meeting of the Metropolitan Water Board last week. The loss was stated to occur under the method of charging 5 per cent. on the rateable value as compared with the old system of supplying by meter. In 1912 on the ninety-six hospitals in its area the Board is said to have lost £9,682. The committee therefore proposes to ask the hospitals to take precautions to keep their consumption of water within bounds. Mr. Musgrave, who is chairman of the committee, stated that the Board was practically subscribing £10,000 a year to the hospitals, and yet that its employees were sometimes refused admission, except on payment of a fee. On the other hand, Mr. Lyell pointed out that an adequate water supply was so essential to the public health that he hoped that no pressure would be put upon the hospitals. The committee's report was in the end approved. In November 1911 a correspondent wrote to us saying: "The Water Board cannot demand to charge a medical charity by measure. When the London Water Bill was under consideration in 1907 the Central Hospital Council approached the Government on the subject, with the result that water is now supplied to hospitals at 5 per cent. on the rateable value." If water is no longer supplied by meter, what reason is there for alleging that hospitals are extravagant in its use? This appears only an underhand way of objecting to a former concession.

### VOLUNTARY AID DETACHMENT DISPLAY.

At the annual War Office inspections of St. John Voluntary Aid Detachments, Numbers 88, 92, 94, and 82, 86, 96, County of London, two interesting displays will take place. At the combined inspection of the first three on June 18, which will be held at St. John's Gate, Clerkenwell, the Chapter House will be fitted up as a complete temporary hospital. On June 21 the chapel of the old Duke of York's School will be fitted up in a similar manner when the other three detachments are inspected. Lady Perrott, Lady Commandant-in-Chief of St. John Voluntary Aid Detachments, will supervise the hospital on the first occasion.

### WOMEN DOCTORS IN INDIA.

Speaking at the annual meeting of the Cambridge Mission to Delhi and the South Punjab recently the Rev. D. Kennedy (Chota Nagpur), organising secretary, Medical Mission Department, Society for the Propagation of the Gospel, referred to his visits to St. Stephen's Medical Mission Hospital at Delhi and St. Elizabeth's Hospital, Karnal. He spoke of the splendid work accomplished by women doctors. It was permissible to doubt whether there was such a great need for so many women doctors in England, but there was absolutely no doubt at all of their need in India. If the people could be first visited in their homes they would be much more likely to go into the hospital for treatment. He would like to see the Delhi and Karnal Hospitals' staffs strengthened, so that they might be able to carry on more of this work. It was very important that they should have the medical work well to the front. Already this work had had the effect in Delhi of winning support from the Government and the attachment of the English in India.

### INFIRMARY SCRUBBERS AND SUPERANNUATION.

The legal advisers to the Southwark Board of Guardians have addressed a letter to the Board dealing with the case referred to in our last issue, in which Judge Parry awarded a scrubber at the infirmary the return of her superannuation contributions. The letter, whilst refraining from commenting on the decision, states that, having regard to the views expressed by the Judge, they should certainly feel unable to advise the Guardians to contest any further actions of a similar character. If, in future, the Guardians desired to avoid the return of the contributions, they would have to consider whether any special arrangement could be entered into with the scrubbers upon their entering into the Guardians' service. As several similar actions are likely to be taken by scrubbers, the matter was referred to the Finance Committee. The unsatisfactory state of the Superannuation Act was again shown in the case of a scrubber who was ill. She suffered from eczema, and it was evident would never be able to continue her work. In order that the Guardians might return her contributions they were compelled to give her a week's notice to terminate her engagement. The Act requires amendment to meet cases like this, and that of the officer who gets married.

### THE HOSPITAL OF ST. JOHN OF JERUSALEM.

THE annual Festival of the Order of the Hospital of St. John of Jerusalem at which all Members and Associates of the Order should be present, will be celebrated on Tuesday, June 24. The annual Commemoration Service will be held at 2.30 P.M. in St. John's Parish Church, Clerkenwell, which comprises the remains of the choir of the ancient Priory Church of the Order. The Bishop of Southwark, Sub-Prelate, will preach the anniversary sermon. The General Assembly will be held at 3.45 in the Chapter Hall at St. John's Gate. Officers and members of all ranks, nursing sisters included, of the St. John Ambulance Brigade and of the Voluntary Aid Detachments of the Territorial Branch who are entitled to be present are requested to appear in uniform.

### ST. THOMAS' HOSPITAL MEDICAL SCHOOL.

PRIZES will be distributed to the students of the Medical School of St. Thomas' Hospital on Tuesday, June 24, at 3 P.M., by Lord Ampthill in the Governors' Hall. A garden party will be held afterwards on the Terrace. The various departments of the hospital and the school will be open to the inspection of visitors.

### TRADE UNION PRINCIPLES AMONG MENTAL HOSPITAL WORKERS.

SIR JOHN JARDINE, M.P., presided at the annual general meeting of the Asylum Workers' Association last week, and a report was presented which laid considerable stress on the unrest in the mental hospital service due to "the active propaganda of trade union principles" among mental hospital employees. The report notes as the first evidence of the awakening of the national conscience to its obligations to mental hospital workers the Asylum Officers' Superannuation Act of 1909, which made the position of mental hospital officers in respect of pensions more definite than before. The fact that compromise with a strong opposition led to the dropping of some of the points during the passage of the Bill led to a fall in the Association's membership, which, however, Sir John Jardine hopes will rally again. Grants of £2 10s. from the Convalescent Fund were made in twenty-eight cases. The Parliamentary Sub-Committee of the Association are alive to the vistas opened up by the Mental Deficiency Bill and are ready to watch the interests of those to be employed in the new service. Dr. Nolan, Down District Mental Hospital, remarks that in Ireland the question of shorter hours is not the real issue, but that the low rates of pay and "the defects inflicted by its opponents on the Asylum Officers' Superannuation Bill of 1909 are the true causes of unrest." The resignation of Dr. Shuttleworth, honorary secretary of the Association since 1897, which was presented last year, is now taking effect. As Sir John Jardine emphasised, mental hospital workers need more defence and protection than other classes because they come little in contact with the community at large. He explained that the provisions of the amending Bill to the Act of 1909 included changes proposing to raise the age of women from twenty to twenty-five, and the concession of a pension, irrespective of age, if they broke down; gratuities to widows and children; the calculation of pension on the last five instead of on the last ten years' service. A resolution supporting the amending Bill was carried unanimously. The moral is that there is a true unionism and a false, and the test of the true is unity and collective strength through a common association.

### LONG SERVICE IN MENTAL HOSPITALS.

AT the annual meeting of the Asylum Workers' Association last week Sir John Jardine was re-elected chairman for the ensuing year, and medals for long and meritorious service were awarded as follows:—Gold medals were presented to Mr. T. Darley, The Retreat, York, with forty years and eight months' continuous service, and to Miss D. J. Johnson, Lancaster County Mental Hospital, with forty years and seven months' continuous service. Silver medals were given to Mr. A. McDonald, Down District, who has served for thirty-eight years and three months, and to Mrs. Neasby, Darenth Colony, with thirty-six years and seven months' service. Bronze medals have also been awarded to Mr. W. H. Leek, Worcester; Mr. D. McArthur, Warford House; Mr. J. G. Lamkin and Mr. T. Neasby, Darenth; Mr. J. Dodkins, Brooke House; Mr. W. Williams, Denbigh; Mr. J. Marshall, Barming Heath; Miss A. A. Mingay and Miss H. H. Church, Colney Hatch.

### PATIENT BATHED IN A VERANDAH.

A RECENT case brings forward again the apparently everlasting question of the management of isolation hospitals. A schoolboy, through his father, sued the Hendon Rural District Council for damages, alleging neglect in his treatment in the Hendon Isolation Hospital. The boy, having developed scarlet fever at school, was conveyed to this hospital. Here, he says, he was compelled to take a bath in a verandah, near a door facing north-east, and where there was a hole in the wall owing to the operations of workmen. Owing to this he stated that he developed rheumatism and heart trouble, which prevents him from entering the Navy. He also complained of neglect in the ward: that there was only one glass for all the patients, and that eggs were always hard-boiled, because there was only one egg-cup for the ward. The defence was that the verandah where the bath was administered was, as nearly as possible, the temperature of the ward, which, however, was not apparently stated. For the rest, the Matron said that there were plenty of glasses and egg-cups on the premises, and it was the fault of the nurse if the ward-supply was not sufficient. The claim was defeated. It seems to us that a verandah can never be the place for a bath to be given to a fever patient. Are there no properly-equipped bathrooms in the Hendon Isolation Hospital? The plea that there were plenty of glasses, etc., on the premises, although the supply in this particular ward was insufficient, appears to be a weak one. Is it not the business of the Matron to see that every ward is properly supplied? The superfluity in one place does not balance the paucity in another. The Hendon Isolation Hospital has escaped without condemnation, but the action clearly was not a wanton one.

### THE RETIREMENT OF A SHEFFIELD CHAIRMAN.

After acting as chairman of the board for twenty-two years Mr. John Marshall is retiring from the Sheffield Royal Infirmary. His portrait has been painted and will be placed in the board-room as a memento of his long and distinguished services to the infirmary. The presentation of the portrait took place last week after a dinner, at which the present chairman, Mr. H. H. Bedford, presided. In an interesting speech the chairman recalled that Mr. Marshall joined the board in 1875 and succeeded Mr. Abram Brooksbank as chairman. During the time in which Mr. Marshall has been a member of the board the hospital's income has risen from £7,000 to £17,000. In the same interval the present out-patients' department, the ophthalmic wards, the new surgical block, the laundry, and the nurses' home have been built. "Mr. Marshall," the chairman proceeded, "has done more for the infirmary, and through the infirmary for Sheffield, than almost any other citizen of our time." Dr. Porter, senior member of the honorary medical staff, gave an enthusiastic tribute to the good relations which Mr. Marshall had preserved between the board and the medical staff, and said that they were parting with a very old friend. An enthusiastic reception greeted Mr. Marshall on his rising to reply. He recalled how clearly the great need for extension had struck him during the early part of his chairmanship, and in referring to the relation between the board and the medical staff, said that he did not think the latter had ever asked for anything which would further the good work without getting it at once, or encouragement for the future. He added that, thanks to Mr. Colin Smith, the convalescent home at Conisborough had been secured, which, though it cost a certain amount of money, was of immense value for good. The portrait is by Miss Ruth Garnett, a London artist.

### THE AGE OF RETIREMENT.

Two illuminated addresses were presented last week at a meeting of the Royal Victoria Infirmary, Newcastle, to Dr. David Drummond, retiring senior honorary physician, who has acted as "honorary" for thirty-four years, and to Sir Thomas Oliver, who is retiring from the post of honorary physician. Dr. Drummond's period of service is second only to that of the late Dr. T. E. Headlam, who was honorary physician for thirty-eight years. Sir Thomas Oliver, in his reply, stated that the age of retirement of hospital physicians in Newcastle was much earlier than in other places. Had the time not come for the committee and governors to consider whether some modification might be made in the age limit? It was a proper thing to give place to younger men, but he asked whether, on the retirement of their physicians and surgeons, five or ten beds should not be offered to them, as was done in Leeds, to retain their interest in the institution. It is not easy to lay down the law on this question, for "consulting physicians and surgeons" in practice of course are not consulted, and thus it would be only the very keen man who really desired to have beds allotted to him.

### SIR THOMAS OLIVER'S REMINISCENCES.

In a speech full of interesting reminiscence, Sir Thomas proceeded to review the strides made in hospital hygiene. The in-patients of the infirmary were better fed, better groomed, and better clad than they were thirty years ago. No longer did they find patients taking dirty jars out of cupboards, and from those jars lifting out, with all sorts of metallic spoons, sour smelling butter, while they cut up their meat with all kinds of cutlery belonging to themselves. It was thought at first, when the changes were brought about, that the expense of the institution would be added to very considerably, but, as experience had since showed, this was not the case. He was convinced, too, that the bright infirmary cutlery, the nice, clean earthenware out of which the food was taken, and the training and instruction given by a body of highly trained nurses could not but have an uplifting influence on the patients passing through the infirmary—an influence which was carried away from the institution and reflected in the home. His above remarks upon the age of retirement are worth further consideration. What are our readers' views?

### MR. ARTHUR JAMES AND CANCER RESEARCH.

As a memorial to his late brother, Mr. William James, Mr. Arthur James has decided to give the income of a sum of £20,000 for cancer research to the Middlesex Hospital, which he singled out for this important gift because here, in his opinion, the clinical and pathological research on the disease and its causation are most intimately associated. It is hardly necessary to remind our readers that the late Mr. William James, to whom this gift is the memorial, was not the famous philosopher and psychologist, but the owner of West Dean Park, near Chichester, who died last year, and at whose house King Edward VII. was accustomed to visit.

### THE MEMORIAL TO MR. JORDAN LLOYD.

The committee of the Queen's Hospital, Birmingham, is appealing for funds for the endowment of one of the surgical wards in the institution as a memorial to the late Mr. Jordan Lloyd, who, as our readers will remember, was honorary surgeon to the hospital for thirty years. If the endowment is able to be carried out the ward will be named after him, and his bust or portrait will be placed in it. Already, we are glad to note,

£500 has been received for this purpose from Mr. W. Waters Butler, while Mr. George Cadbury, Mr. Dudley Docker, Mr. T. Grosvenor Lee, and Mr. H. Mitchell have given £100 each. Mr. Jordan Lloyd is well worth commemorating, and the Queen's Hospital badly needs endowment if it is not merely to keep abreast of modern development but even to make the fullest use of the facilities already at its disposal. Either of the above, the late surgeon or the institution, should attract the support not merely of present Birmingham men but of all who have cause to remember the work of both.

### A RETROGRESSIVE NEW INFIRMARY.

A NEW infirmary is to be built for the Holywell Board of Guardians at a cost of £7,000, and at a recent meeting a member called attention to the fact that the plans did not show either an operation room or a mortuary. The Chairman, Mr. James Prince, stated in reply that the plans had been prepared under the observation of the Local Government Board, and had an operation room been necessary they would no doubt have provided it. Mr. Prince appears to forget that it is quite as much the duty of Guardians to make known their needs to the Local Government Board as for the Board to insist upon a standard of administration. We can imagine no circumstances counterbalancing the need for every up-to-date infirmary to have an operation room and a mortuary, and do not envy the doubtful distinction which must attach to the new Holywell institution so long as it is unprovided for in these respects. The smallness of the infirmary is no sufficient excuse for inadequate equipment, since it remains a unit and one that is likely to grow.

### METROPOLITAN HOSPITAL SUNDAY FUND.

THE General Purposes Committee have had under consideration the appointment of a successor to the late Sir Edmund Currie in the office of secretary. The Fund is fortunate in having on its staff two gentlemen, Mr. J. A. R. Lander, who has been in the office of the Fund for twenty-five years, and Mr. Arnold James, Mr. Owthwaite's successor, who has held the post of statistical officer for ten years. Both have rendered excellent services in their respective offices, and are well known to those who have to do with the Fund and its work. They have also won the entire confidence of the chairmen of committees and of the honorary officers of the Fund. The General Purposes Committee have accordingly unanimously elected Mr. Arnold James as secretary, and Mr. J. A. R. Lander as assistant secretary to the Fund.

### THE RIGHTS AND WRONGS OF DRASTIC PROHIBITIONS.

THE interesting case of Eastes *v.* Russ we deal with on another page. Here we merely wish to add that we cannot imagine how any really competent bacteriologist with the least spark of ambition in his nature could, with open eyes, consent to such an all-life inhibition of his energies as that involved in a lifelong prohibition to practise outside the laboratory in which he had been once employed. Therefore, we sympathise with the defendant in his successful plea. But we do feel disposed to agree with the Judge that the plaintiff does suffer some moderate degree of hardship by the result of the trial. The restrictions he sought to impose on Dr. Russ were very stringent indeed, and we cannot help thinking that a less drastic prohibition, more clearly expressed, would have been a better policy than that of clause 12. Dr. Eastes' construction of the latter may not be unenforceable in law, but it would be so harsh and unconscionable that we feel, as Mr. Justice Sargant said he too did, very glad that it was set aside on other grounds.

### STAFF CHANGES AT BERMONDSEY.

THE Bermondsey Board of Guardians are considering a scheme for the thorough reorganisation of their medical staff, both in the institutions and for the treatment of the outdoor poor. Two recent deaths enabled the Board to grapple with the question at the present time. Dr. Alfred Salter, J.P., who has been elected a Guardian, has formulated a scheme which will result in Dr. E. S. Bell, the present medical superintendent at the infirmary, being appointed chief medical officer to the board, with the supervision of the two workhouses and of the five medical out-relief districts. In addition, the assistant medical staff at the infirmary is to be increased from two to five, who are to act as deputies at the workhouses and the out-relief districts. Members of the present medical staff are to be invited to fill the positions; advertisements to be issued for the vacancies. Dr. Salter, while agreeing that the total cost of the medical staff would probably be somewhat increased, believes that the alterations would greatly improve the treatment for the sick poor.

### THIS WEEK'S DRUG MARKET.

WITH the exception of a good demand for seasonable articles, such as citric acid, cream of tartar, and essence of lemon, business in the Drug market continues quiet. Citric acid has advanced in price, and, with a continuance of hot weather, prices are likely to advance still further. Extremely high prices are quoted for essence of lemon, and cream of tartar is also dearer. Menthol still has a downward tendency in value. Balsam tolu continues scarce, as also does cascarilla. Cantharides are dearer. Galbanum tends lower in value. Reports of the coming opium crop are favourable, and prices tend lower; morphine has also a slightly lower tendency in price. Gentian root is rather dearer. Senega root is scarce and dearer. The price of saffron is not so firmly maintained. There has been no further development in the Quinine market. It is thought probable that cod-liver oil may shortly commence to move upwards in value, but at present the demand is very small.

